# Influence of weather and seasonal factors on whitefly dynamics, associated endosymbiotic microbiomes, and *Begomovirus* transmission causing tomato leaf curl disease: insights from a metagenomic perspective

**DOI:** 10.3389/fmicb.2025.1555058

**Published:** 2025-03-12

**Authors:** S. Sujatha, Kopparthi Amrutha Valli Sindhura, Prasanna S. Koti, Shridhar Hiremath, Mantesh Muttappagol, H. D. Vinay Kumar, K. S. Shankarappa, V. Venkataravanappa, K. M. Srinivas Reddy, C. N. Lakshminarayana Reddy

**Affiliations:** ^1^Department of Agricultural Entomology, College of Agriculture, University of Agricultural Sciences, GKVK, Bangalore, India; ^2^Department of Biotechnology, College of Agriculture, University of Agricultural Sciences, GKVK, Bangalore, India; ^3^Centre of Infectious Diseases, Biological Sciences and Technology Division, CSIR-North East Institute of Science and Technology, Jorhat, India; ^4^Department of Plant Pathology, College of Agriculture, University of Agricultural Sciences, GKVK, Bangalore, India; ^5^Department of Plant Pathology, College of Horticulture, University of Horticultural Sciences, Bagalkot, Bengaluru, India; ^6^Division of Crop Protection, ICAR-Indian Institute of Horticultural Research, Hessaraghatta Lake PO, Bangalore, India

**Keywords:** *Bemisia tabaci*, metagenomics, population dynamics, cryptic species, Proteobacteria, tomato leaf curl virus disease, tomato disease management, weather parameters

## Abstract

**Introduction:**

*Bemisia tabaci* (Gennadius) is a globally significant agricultural pest, responsible for transmitting over 120 plant viruses, including those from the Begomovirus genus, which contribute to considerable crop losses. The species complex comprises cryptic species, associated with a diverse array of bacterial endosymbionts that play essential roles in host nutrition, virus transmission, and overall host adaptability. These endosymbionts are classified into primary and secondary categories, with primary endosymbionts forming obligatory, long-term associations, and secondary endosymbionts influencing factors such as biotype differentiation and vector competency. Notably, these microbial communities enhance *B. tabaci*’s capacity to transmit viruses, including the tomato leaf curl virus (ToLCuV), which poses a significant threat to tomato production.

**Methods:**

In this study, we examined the population dynamics of *B. tabaci* across three major tomato-growing regions in Karnataka, South India, focusing on their seasonal associations with endosymbionts and the incidence of tomato leaf curl disease (ToLCuD). Multiple regression analysis was employed to assess the influence of weather parameters on whitefly populations and disease prevalence. Additionally, we constructed a metagenomic profile to evaluate the effects of geographical location, seasonality, environmental factors, and agricultural practices on the bacterial communities associated with *B. tabaci*. Species-specific primers were used to validate the presence and diversity of these bacterial communities.

**Results:**

Meteorological data revealed a positive correlation between temperature and *B. tabaci* populations, which corresponded with an increased incidence of ToLCuD. Genetic characterization of the whitefly identified Asia II-5 and Asia II-7 cryptic species as the dominant forms in the surveyed regions, with Portiera emerging as the most prevalent endosymbiont. A more in-depth analysis of the microbial communities associated with *B. tabaci*, utilizing 16S rRNA metagenomic sequencing, revealed a dominance of the Proteobacteria phylum. The endosymbiotic bacterial consortium was primarily composed of *Candidatus Portiera*, *Candidatus Hamiltonella*, *Candidatus Rickettsia*, and *Candidatus Arsenophonus*.

**Discussion:**

The metagenomic analysis revealed a highly diverse array of bacterial communities, with 92% of sequences classified under Proteobacteria, representing a spectrum of microbial types associated with *B. tabaci* ranging from parasitic and pathogenic to mutualistic. Within this phylum, Alphaproteobacteria were predominant, known for their role as facultative symbionts, while Gammaproteobacteria provided essential nutrients to arthropods, enhancing their survival and fitness. The interplay of continuous and intensive tomato cultivation, elevated temperatures, favorable host plants, and abundant viral inoculum creates an ideal environment for the proliferation of *B. tabaci* and the widespread transmission of ToLCuD. The presence of diverse cryptic species of *B. tabaci*, which are efficient viral vectors, further complicates the situation. These findings underscore the urgent need for integrated management strategies globally to control both whitefly populations and ToLCuD, ensuring the protection of tomato crops and the sustainability of farmer livelihoods.

## Introduction

*Bemisia tabaci* (Gennadius) (Hemiptera: Aleyrodidae), a species complex comprising at least 46 cryptic species, is morphologically indistinguishable yet reproductively isolated (De Barro et al., 2011; Boykin et al., 2013; Rehman et al., 2021). This pest significantly impacts various crop species through phloem feeding and the transmission of over 120 plant viruses, primarily from the *Begomovirus* genus within the *Geminiviridae* family (Gilbertson et al., 2015; Polston et al., 2014). By causing extensive damage to economically important plants and contributing to substantial agricultural losses globally, *B. tabaci* underscores its role as a major vector of persistently transmitted begomoviruses.

In natural ecosystems, many insects host diverse microorganisms, including endosymbiotic bacteria (Gosalbes et al., 2010). Among these, *B. tabaci* exhibits the highest diversity of bacterial associations, classified into primary and secondary endosymbionts based on their role and relationship with the host (Gnankiné et al., 2013; Gosalbes et al., 2010). Primary endosymbionts (PEs) establish obligatory, long-term associations, co-evolving with their insect hosts. In contrast, secondary endosymbionts (SEs) are more recently evolved, occasionally migrating horizontally between hosts and residing in the hemolymph without being obligatory (Mahadav et al., 2008; Himler et al., 2011; Kliot et al., 2014). As a phloem-feeding Homoptera, *B. tabaci* harbors a range of endosymbionts that fulfill various functional and nutritional roles. These include carotenoid provision (Sloan and Moran, 2012) and essential nutrients such as B-complex vitamins and amino acids from obligate endosymbionts like *Portiera aleyrodidarum* (Xie et al., 2018). Additionally, endosymbionts assist in virus transmission; for instance, a GroEL homolog from these bacteria supports the circulative transmission of viruses by shielding them from degradation in the haemolymph (Morin et al., 1999; Rana et al., 2012). Secondary endosymbionts are crucial for biotype differentiation, transmission potential, heat tolerance, pesticide resistance, and overall adaptability of *B. tabaci*, enhancing its status as a significant global pest. For example, *Hamiltonella* sp., found in the B-biotype of *B. tabaci*, facilitates the spread of the tomato yellow leaf curl virus (TYLCV) (Gottlieb et al., 2010). Recent studies also highlight that bacterial endosymbionts influence viral-vector interactions in a tripartite manner (Seruwagi et al., 2018). Given the variability among cryptic species and their endosymbionts, understanding genetic diversity is crucial for identifying vulnerabilities and developing more effective pest management strategies.

Traditional methods for exploring the genetic diversity of *B. tabaci* and its associated bacteria, such as culturing isolates (Bauer et al., 2015; Bolaños et al., 2016), 16S rRNA gene amplification with specific primers (Osei-Poku et al., 2012; Shi et al., 2016), and constructing 16S rRNA gene clone libraries (Jones et al., 2013), have proven inadequate in uncovering the full genetic diversity of polymicrobial communities associated with whiteflies. The advent of next-generation sequencing (NGS) technologies has revolutionized this field. Metagenomics, a powerful NGS-based approach, now enables comprehensive genotypic characterization and provides a robust framework for delineating species boundaries and assessing the genetic diversity of entire bacterial communities associated with *B. tabaci* (Gibbons et al., 2009; Thomas et al., 2012).

Tomato (*Solanum lycopersicum* L.) is a vital vegetable crop cultivated across diverse agro-climatic zones in the country. With an annual production of 208 lakh tonnes and a yield of 27.73 tonnes per hectare, it ranks as the second most significant vegetable crop globally, spanning an area of 8,12,000 hectares (Mane et al., 2022). However, tomato production has been facing substantial global challenges in recent decades due to numerous viral diseases, particularly tomato leaf curl disease (ToLCuD) caused by tomato leaf curl virus (ToLCV) belonging to the *Begomovirus* genus and *Geminiviridae* family, which has led to over 90% yield loss in Karnataka State (Saikia and Muniyappa, 1989). The introduction of the B-biotype whitefly to India in the late 1990s exacerbated this issue, increasing the incidence of whitefly-transmitted viral diseases in various vegetable crops by 3–4 times. This surge in disease prevalence has severely affected tomato cultivation in the region, often leading to crop abandonment due to the difficulty in managing these viral infections (Banks et al., 2001).

To address these issues, we investigated the population dynamics of whiteflies across six regions in major tomato growing locations of Karnataka, South India and their seasonal association with endosymbionts and incidence of ToLCuD. We employed multiple regression analysis to examine the impact of weather parameters on whitefly populations and disease prevalence. To assess the influence of geographical location, seasonality, environmental factors, and agricultural practices on bacterial communities, we constructed a metagenomic profile. This was further validated using species-specific primers to analyse and confirm the bacterial communities associated with *B. tabaci* populations.

Tomato cultivation in Karnataka’s eastern dry zone, characterized by specific climatic, topographical, and agricultural conditions, supports substantial whitefly populations. The whitefly gut’s relative isolation and the intensive farming of particular plant species influence bacterial colonization. Thus, semi-arid climates could be pivotal for studying natural whitefly populations and their associated endosymbionts. We utilized next-generation sequencing of 16S rRNA gene from the gut microbiome to examine the relative abundance of various bacterial endosymbionts in *B. tabaci* across different geographic settings.

The findings from this study are expected to provide comprehensive insights into the dynamics of whitefly populations, associated endosymbionts and the seasonal incidence of ToLCuD. Additionally, the research will enhance our understanding of the entire bacterial community, including PEs and SEs associated with whiteflies, and their variation in relation to geographic location, seasonal changes, environmental conditions, and agricultural practices.

## Materials and methods

### Area of study for monitoring *Bemisia tabaci* population and ToLCD incidence

Between 2019 and 2021, systematic monthly surveys were conducted in tomato fields across the Eastern Dry Zone of Karnataka, South India, to evaluate the seasonal dynamics of *B. tabaci*, incidence of ToLCuD, and environmental influences. These surveys also facilitated the collection of *B. tabaci* samples for metagenomic analysis of their associated endosymbiont populations. The study encompassed six locations across three districts: Doddaganjur and Kencharlahalli in Chikkaballapur district, Kalluru and Srinivaspur in Kolar district, and Tippuru and Kundana in Bangalore Rural district.

### Recording *Bemisia tabaci* population

Whitefly abundance was monitored at monthly intervals across three consecutive growing seasons *viz*., Rabi (October to January), Summer (February to April), and Kharif (July to September) at designated locations within tomato fields. To assess whitefly populations, tomato plants were randomly selected using a zigzag pattern throughout the field. Adult whiteflies were enumerated from the top, middle, and bottom leaves of 10 randomly chosen plants per field during the morning hours when whitefly activity is minimal. The mean population data for each field were then calculated to determine seasonal and spatial variations in whitefly abundance.

### Recording incidence of tomato leaf curl disease

To estimate the incidence of leaf curl disease, 10 micro-plots of size 10 × 10 m /acre of tomato fields were randomly selected. The incidence was assessed by counting both the number of infected plants and the total number of plants within each micro-plot. The mean incidence value was then calculated for each field based on these counts.

The percent disease incidence was calculated using the formula below:

$\mathrm{Percent disease incidence}=\left[\begin{array}{l} \mathrm{Number of diseased}\;\\ \mathrm{plants}/\mathrm{Total number}\;\\ \mathrm{of plants examined} \end{array}\right]\;X\;100$

To examine the impact of meteorological variables on the incidence of whiteflies and ToLCuD, data on weather parameters *viz*., maximum and minimum temperatures, morning and afternoon relative humidity, sunshine hours, and total rainfall were collected from the nearest University of Agricultural Sciences, Bangalore meteorological stations. Subsequently, correlation and regression analyses were performed to assess the relationships between whitefly populations, the percentage of plants exhibiting leaf curl symptoms, and the recorded weather parameters.

### Molecular characterization of *Bemisia tabaci* and metagenomics sample preparation

*Bemisia tabaci* samples were collected for the identification of cryptic species and associated endosymbionts between October 2019 and April 2021.Adult whiteflies were individually preserved in microcentrifuge tubes containing 95% (v/v) ethanol and stored at −20°C. Cryptic species were identified through genetic barcoding of the mitochondrial cytochrome oxidase I (*mtCOI*) gene sequence, with biotypes confirmed against reference sequences from the NCBI database, maintaining a 3.5% genetic divergence threshold (Dinsdale et al., 2010). A total of nine pooled genomic samples of *B. tabaci* were used for the metagenomics analysis based on location and season; three genomic pooled samples representing each district ONCHI, DJCHI, FMACHI from Chikkaballapur (CHI), ONKOL, DJKOL, FMAKOL from Kolar (KOL) and ONBRU, DJBRU, and FMABRU from Bangalore Rural (BRU). Within the district, three samples represent three different seasons (October–November, December–January and February, March and April) during which collection was made (Supplementary Table S1). Metagenomic analysis was conducted to assess variations in endosymbiont associations across different locations in tomato fields.

### Isolation of DNA from adult *Bemisia tabaci*

Total genomic DNA was extracted from *B. tabaci* samples using a modified Chelex 100 method (Rua et al., 2006). Adult whiteflies were surface-sterilized sequentially with sterile distilled water, 70% ethanol, and 0.1% sodium hypochlorite. Each whitefly was then homogenized in 100 μL of TE buffer solution containing 5% Chelex 100 resin and 300 μg of Proteinase K. The homogenate was centrifuged at 13,000 rpm for 10 min, and the upper aqueous supernatant containing the DNA was transferred to a fresh tube and stored at −80°C for further analysis. DNA quality and quantity were assessed using a Nanodrop spectrophotometer and agarose gel electrophoresis.

### Amplification and sequencing of the *Bemisia tabaci* *mtCOI* gene and the 16S rRNA gene of bacterial endosymbionts

The identification of *B. tabaci* species in the collected samples was achieved through molecular techniques involving DNA extraction using modified Chelex 100 method, amplification and sequencing of the *mtCOI* gene to genetically characterize the species. For metagenomic sampling, the 16S rRNA gene was amplified from genomic DNA using polymerase chain reaction (PCR) with universal primers. The forward primer (AGAGTTTGATCMTGGCTCAG) and reverse primer (TACGGYTACCTTGTTACGACTT) were employed in the amplification process. The reaction mixture comprised 0.2 μL of template DNA (20 ng), 0.1 μL of each primer (10 pM/μL), 1 μL of 10 mM dNTPs, 0.2 μL of Taq DNA polymerase (TaKaRa Taq 250 U Cat# R001A), 2.5 μL of Taq DNA buffer A, and 15.9 μL of grade I water. The PCR thermal cycling protocol included an initial denaturation at 94°C for 2 min, followed by 29 cycles of denaturation at 94°C for 45 s, primer annealing at 55°C for 1 min, and extension at 72°C for 2 min, with a final extension at 72°C for 10 min. The amplified products were resolved by electrophoresis on a 0.8% agarose gel. These amplicons were eluted from the gel and were sequenced at Eurofins Genomics, Bangalore, India.

### Metagenomic sequencing and analysis of *Bemisia tabaci* samples

DNA from nine samples (ONCHI, ONKOL, ONBRU, DJCHI, DJKOL, DJBRU, FMACHI, FMAKOL, and FMABRU) was sequenced targeting the V4 region of the 16S rRNA gene from whitefly samples using Illumina MiSeq amplicon sequencing technology, resulting in 2×250 base pair paired-end libraries. The acquired raw reads were processed using the DADA2 (v 1.18) software package (Callahan et al., 2016). Quality profiles of forward and reverse reads were assessed before and after filtering using the *plotQualityProfile* function. Filtering and trimming were performed with the *filterAndTrim* function, resulting in gzipped fastq files. Reads were truncated to 225 nucleotides for forward reads and 200 nucleotides for reverse reads, and those with ambiguous nucleotides (N) and PhiX control reads were removed.

The *learnErrors* function was used to estimate sample error rates and characterize sequence variants. Paired-end reads were combined into a comprehensive denoised sequence using the *mergePairs* function. The *makeSequenceTable* function created an Amplicon Sequence Variant (ASV) table, and chimeric sequences were removed with the *removeBimeraDenovo* function. Taxonomic assignment was conducted using the DECIPHER program (Murali et al., 2018), with SILVA v138 as a reference. ASV taxonomic classifications were made at various taxonomic levels using the IdTaxa tool.

Richness and diversity were evaluated using rarefaction curves using the *rarecurve* function from the vegan package (Oksanen et al., 2001) and Shannon’s and Simpson’s diversity indices utilizing the *plot_richness* function from the phyloseq package (McMurdie and Holmes, 2013). Euclidean distances and hierarchical clustering were computed using the *dist* and *hclust* functions in R. Multidimensional scaling and Principal Coordinates Analysis (PCoA) were performed using the *ordinate* function from the phyloseq package to assess sample-relatedness and generate PCoA plots. Taxonomic classifications at the phylum, class, family, and genus levels were visualized using the *plot_bar* function (Toutenburg, 1981).

### Validation of whitefly, *Bemisia tabaci* symbionts

The primary and secondary endosymbionts identified through metagenomic analysis of nine samples were further validated using species-specific primers (Table 1). The PCR products were electrophoresed on 0.8% agarose gels stained with ethidium bromide and visualized using a gel documentation system (Molecular Imager BIORAD, California, United States).

**Table 1:** List of species-specific primers used to investigate the association of primary and secondary endosymbionts in *Bemisia tabaci.*

Organism	Gene amplified	Sequence (5’-3’)	Annealing temp. (°C)	Product size (bp)	References
*Portiera*	16S rRNA	Por-F: TGCAAGTCGAGCGGCATCAT Por-R: AAAGTTCCCGCCTTATGCGT	56	1,000	Zchori Fein and Brown (2002)
*Hamiltonella*	16S rRNA	Ham-F: TGAGTAAAGTCTGGAATCTGG Ham-R: AGTTCAAGACCGCAACCTC	52	700	Su et al. (2014)
*Wolbachia*	16S rRNA	Wol-F: CGGGGGAAAAATTTATTGCT Wol-R: AGCTGTAATACAGAAAGTAAA	55	700	Nirgianaki et al. (2003)
*Arsenophonus*	23S rRNA	Ars-F: CGTTTGATGAATTCATAGTCAAA Ars-R: GGTCCTCCAGTTAGTGTTACCCAAC	56	600	Thao et al. (2003)
*Cardinium*	16S rRNA	Car-F: GCGGTGTAAAATGAGCGTG Car-R: ACCTMTTCTTAACTCAAGCCT	52	400	Zchori Fein and Brown (2002)
*Rickettsia*	16S rRNA	Rick-F: GCTCAGAACGAACGCTATC Rick-R: GAAGGAAAGCATCTCTGC	58	900	Gottlieb et al. (2006)

## Results

### Monitoring of *Bemisia tabaci* population and incidence of tomato leaf curl disease

**Figure 1:**
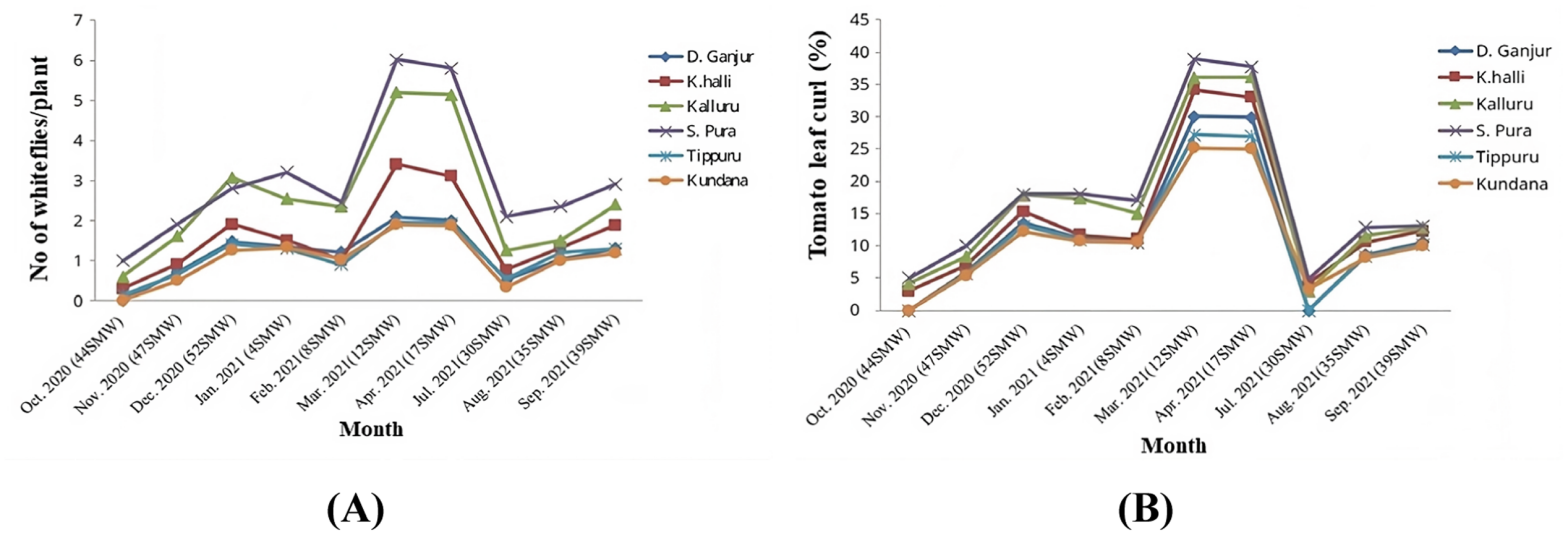
Population dynamics of *B. tabaci* and incidence of tomato leaf curl disease in the study area comprised of three districts with two locations each. Doddaganjur and Kencharlahalli from Chikkaballapur, Kalluru and Srinivaspura from Kolar and Tippuru and Kundana from Bangalore rural districts **(A)** Whitefly density per plant, **(B)** Percentage incidence of tomato leaf curl disease.

Data on the population dynamics of *B. tabaci* and the incidence of tomato leaf curl disease (ToLCuD) were collected over a 10-month period from 2020 to 2021 across three districts in the major tomato-growing regions of Karnataka State. A seasonal pattern was observed, with an increase in the number of insects per plant during the summer months of March and April (Figure 1A), there existed a subsequent rise in the percentage incidence of ToLCuD (Figure 1B). The results obtained are presented in Supplementary Table S2, which revealed that the average whitefly population was 1.75 per 10 plants, with an overall ToLCuD incidence of 13.71%.

In Kolar district, the highest whitefly populations were recorded in Srinivaspur (3.05/10 plants) and Kalluru (2.56/10 plants), with highest leaf curl incidence of 13.04 and 12.80%, respectively. In Chikkaballapur district, Kencharlahalli and Doddaganjur had moderate whitefly populations and leaf curl incidence, with whitefly counts of 1.60 and 1.16 per 10 plants, and disease incidences of 12.30 and 10.60%, respectively. The lowest whitefly population was observed in locations of Bangalore Rural districts, Tippuru and Kundana, with 1.12 and 1.03 whiteflies per 10 plants and leaf curl incidences of 10.20 and 10.00%, respectively.

Since seasonal variations can influence the whitefly population dynamics and virus disease incidence, data recorded was analysed in this regard as well. During the Rabi season of 2020–21, whitefly population ranged from 0.00 to 1.47 per 10 plants, peaking during the 52^nd^ Standard Meteorological Week (SMW). Leaf curl incidences during this period varied from 0.00 to 13.49%. In the summer of 2021, whitefly population peaked at 2.08 per 10 plants, and leaf curl disease incidence reached 30.01% during the 12^th^ SMW. The Kharif season of 2021 showed lower overall whitefly population and disease incidence, with 1.30 whiteflies per 10 plants and 10.60% leaf curl incidence during the 39^th^ SMW.

The study indicates the seasonal variation in the whitefly population and ToLCD incidence in the major tomato-growing areas of Karnataka State. These findings highlight the need for targeted pest and disease management strategies in these high-impact areas.

### Effect of weather parameters on the incidence of whitefly population and ToLCuD

This study investigated the relationship between whitefly population and weather parameters using Multiple Linear Regression (MLR) (Supplementary Table S3). Analysis across villages in Chikkaballapur district revealed consistent trends between whitefly populations and weather variables. A positive correlation was observed with maximum temperature (*r* = 0.62 to 0.84*), while negative correlations were found with minimum temperature (*r* = −0.27 to 0.01), maximum relative humidity (*r* = −0.55 to −0.80*), minimum relative humidity (*r* = −0.63 to −0.74*), and rainfall (*r* = −0.52 to −0.69*). Regression models indicated that increased temperature generally led to higher whitefly population, whereas lower humidity and rainfall were associated with reduced population. Weather factors collectively influenced 70 to 93% of the variability in whitefly population across the villages considered for the study. A similar trend was observed in all the locations.

Similarly, ToLCuD exhibited consistent patterns relative to weather variables. ToLCuD showed a positive correlation with maximum temperature (*r* = 0.61 to 0.84*) and negative correlations with minimum temperature, relative humidity, and rainfall. The impact of the *B. tabaci* population on ToLCuD was mixed, with varying positive and negative correlations depending on the village location. Regression models indicated that increase in temperature and *B. tabaci* population often resulted in higher incidences of ToLCuD, while reductions in other weather variables generally decreased disease prevalence. Collectively, weather factors accounted for 80 to 99% of the variability in ToLCuD across the villages.

### Molecular characterization of *Bemisia tabaci*

Genetic analysis and barcoding of the mitochondrial cytochrome oxidase 1 (*mtCO1*) gene revealed two predominant cryptic species of *B. tabaci* in tomato fields from Karnataka: Asia II-5 and Asia II-7. Biotype identification was confirmed by comparing with reference sequences from the NCBI database. The results indicated that whitefly samples from Chikkaballapur included both Asia II-5 and Asia II-7 biotypes, whereas samples from Kolar and Bangalore Rural were exclusively of the Asia II-5 biotype.

### Metagenomic analysis of *Bemisia tabaci* samples

To accurately assess bacterial diversity across evolutionary lineages of *B. tabaci* cryptic species, we employed the Illumina sequencing platform on nine samples (DJBRU, DJCHI, DJKOL, FMABRU, FMACHI, FMAKOL, ONBRU, ONCHI, and ONKOL) collected from different seasonal periods and locations. This approach generated a total of 195,382, 224,398, 223,258, 200,126, 145,178, 207,794, 187,222, 191,938, and 201,532 paired-end reads for each respective sample (Supplementary Table S4). Integrating these 1,776,828 reads, we identified 3,748 Amplicon Sequence Variants (ASVs). After removing singletons and chimeric sequences, 33% of these ASVs (1,219) were confirmed as authentic.

### Composition of the *Bemisia tabaci’*s bacterial community

**Figure 2:**
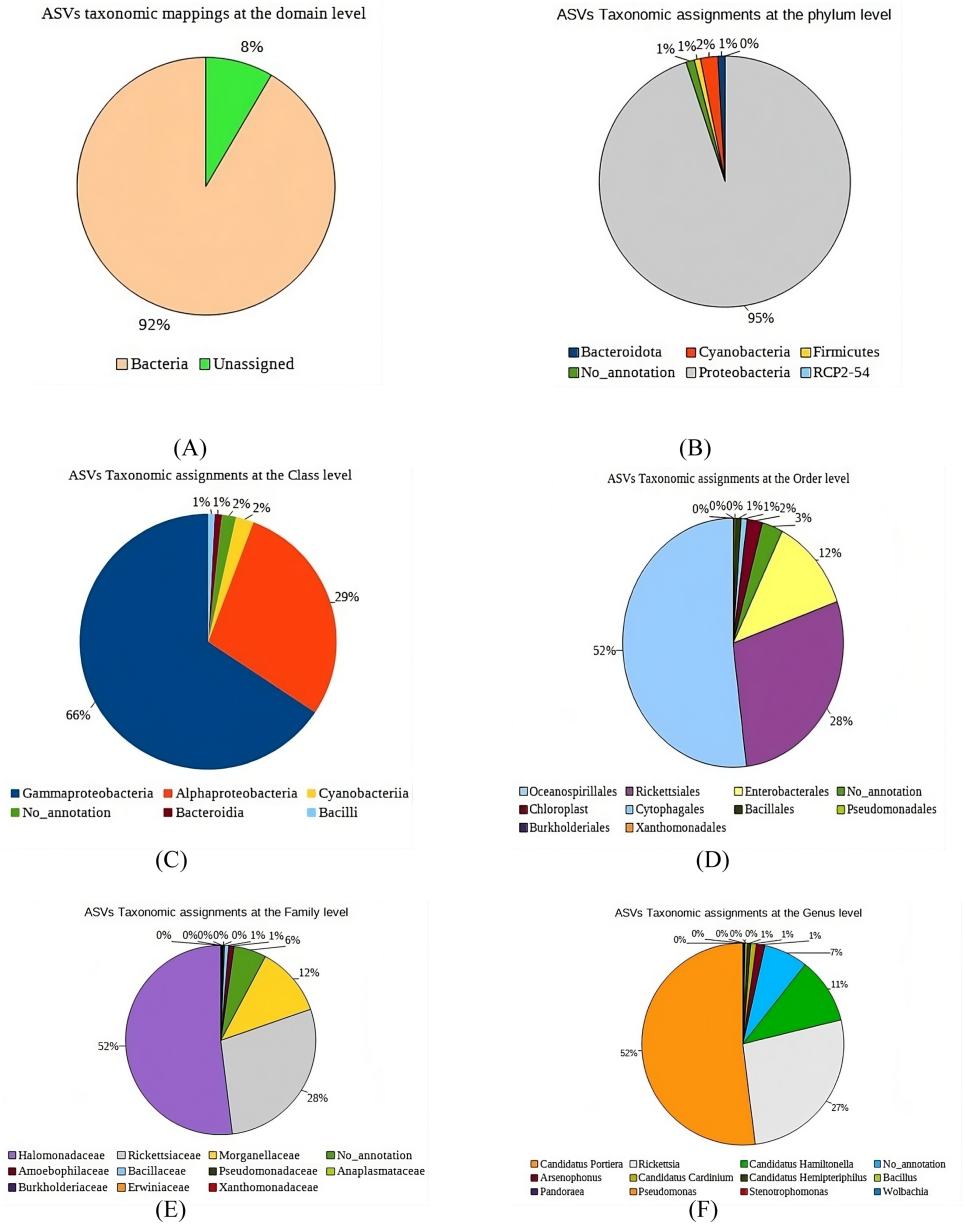
Microbiome composition of *B. tabaci* samples collected from three tomato growing regions of Karnataka at Various Taxonomic Levels: **(A)** Domain, **(B)** Phylum, **(C)** Class, **(D)** Order, **(E)** Family, **(F)** Genus.

At the domain level, 92% of the ASVs from *B. tabaci* were classified as bacteria, with 8% remaining unassigned (Figure 2A). Within the bacterial ASVs, Proteobacteria dominated at 95% of the total phylum-level diversity (Figure 2B). This group was further divided, with 66% identified as Gammaproteobacteria, 29% as Alphaproteobacteria, and 2% as Cyanobacteria. Bacilli and Bacteroidia each represented 1%, with an additional 1% of ASVs remaining unclassified at the class level (Figure 2C). At the order level, the majority of ASVs were classified into Oceanospirillales (52%), Rickettsiales (28%), and Enterobacteriales (12%), while Pseudomonadales, Rickettsiales, and Cytophagales each contributed 1–3% (Figure 2D). Family-level analysis revealed three predominant families (Figure 2E): Morganellaceae (11%), Rickettsiaceae (28%), and Halomonadaceae (52%). At the genus level, *Candidatus Portiera* was the most abundant (52%), followed by *Rickettsia* (27%) and *Candidatus Hamiltonella* (11%) (Figure 2F).

We also observed a direct correlation between the number of endosymbionts detected and the percentage of disease incidence, rather than the population of whiteflies. For instance, the highest disease incidence of 38.10% corresponds to the detection of 11 endosymbionts at the genus level, while the lowest disease incidence of 2.75% is associated with the detection of 8 endosymbionts (Table 2).

**Table 2:** Whitefly population, percentage of disease incidence, and number of endosymbionts detected in the respective samples.

Sample name	Population of whiteflies	% ToLCD	Endosymbionts detected (Genus level)
ONCHI	0.63	5.23	9
ONKOL	1.275	6.87	9
ONBRU	0.38	2.75	8
DJCHI	1.55	38.10	11
DJKOL	2.9	17.8	9
DJBRU	1.31	11.72	7
FMACHI	2.13	24.771	10
FMAKOL	4.49	30.13	9
FMABRU	1.59	20.89	6

### Seasonal and location wise bacterial composition

**Figure 3:**
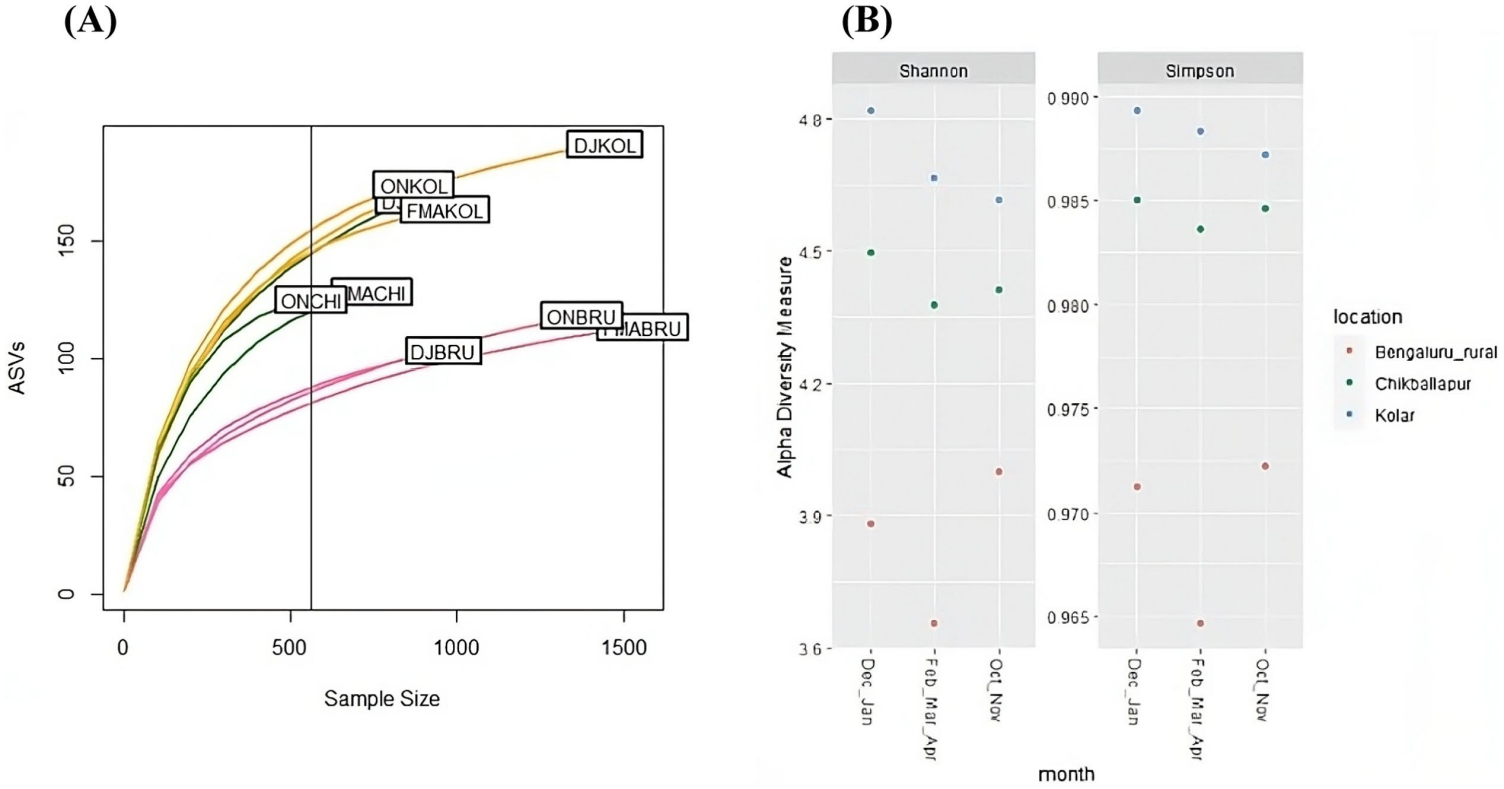
Alpha diversity plots: **(A)** Rarefaction curves illustrating the sequencing depth of *B. tabaci* samples collected from different locations across various seasons; **(B)** Shannon and Simpson diversity indices for samples collected from different locations during different seasons.

The rarefaction curve analysis demonstrated that samples from Kolar and Chikkaballapur exhibited greater bacterial diversity compared to those from Bangalore Rural (Figure 3A). Specifically, the Bangalore Rural samples showed lower sampling depth and richness during the December–January period relative to those from Kolar and Chikkaballapur during the same season. Despite these differences, richness trends were consistent across all sites when categorized by season. Notably, Kolar and Chikkaballapur samples displayed higher species richness and evenness, as indicated by Shannon and Simpson indices, compared to Bangalore Rural samples, particularly evident from February through April (Figure 3B).

The ordination plots revealed a clear separation between Bangalore Rural and the other locations (Supplementary Figure S1A), while the month-wise ordination plot showed overlapping sample distributions (Supplementary Figure S1B). Hierarchical clustering confirmed that samples from Bangalore Rural were more closely related to each other, whereas samples from Chikkaballapur showed greater diversity and correlation with Kolar samples (Supplementary Figure S1C). Preliminary analyses suggest that microbial communities from Kolar and Chikkaballapur are more similar to each other than to those from Bangalore Rural.

**Figure 4:**
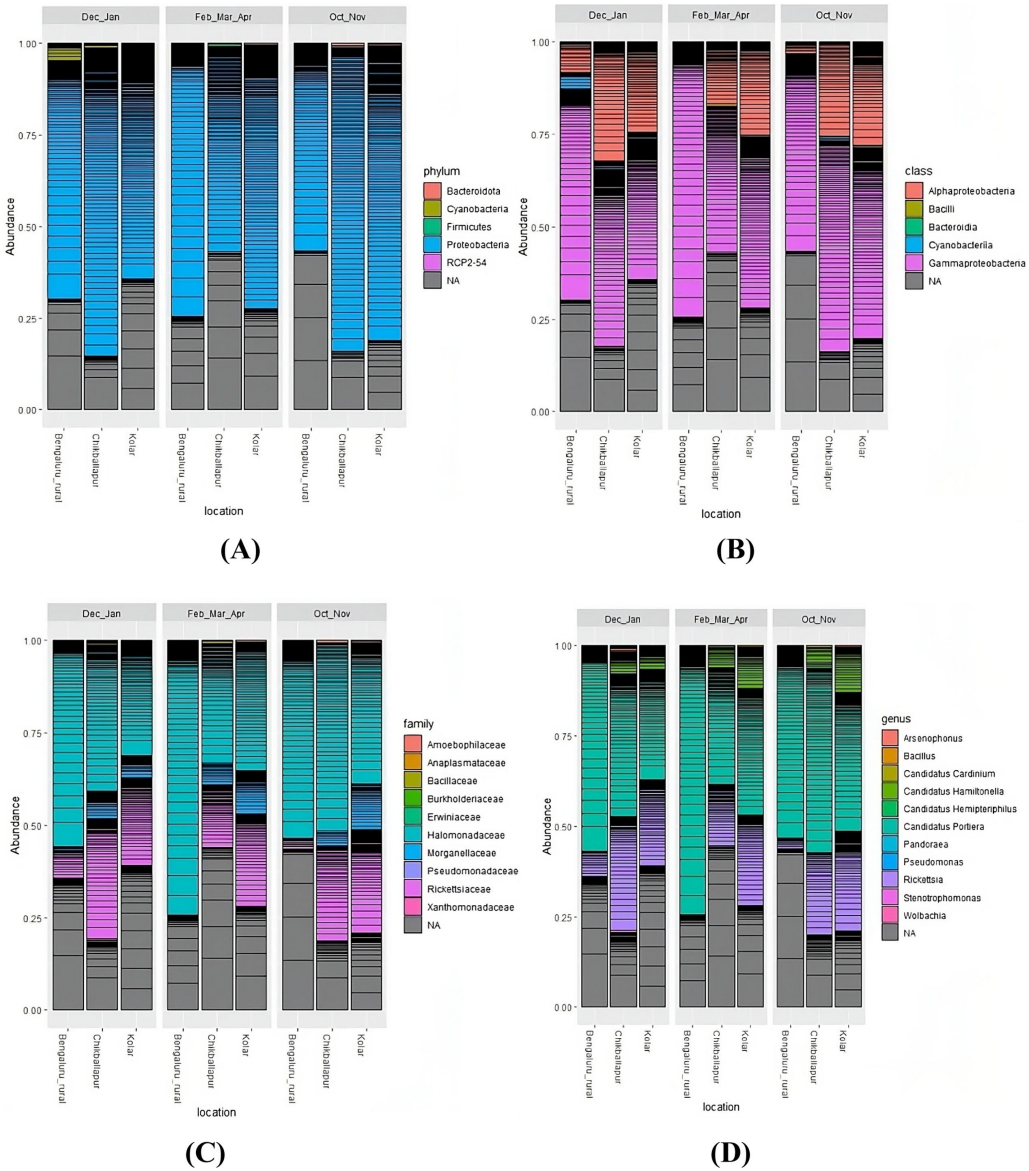
Bar plots illustrating the taxonomic classification and abundance of amplicon sequence variants from the metagenomic data obtained from *B. tabaci* at the following levels: **(A)** Phylum, **(B)** Class, **(C)** Family, and **(D)** Genus.

Taxonomic classifications of bacterial communities across different seasons and locations are depicted in abundance bar plots. At the phylum level, Proteobacteria predominated (Figure 4A), with further subdivision into Alpha- and Gamma-proteobacteria at the class level (Figure 4B). At the family level, Halomonadaceae and Rickettsiaceae were the most prevalent (Figure 4C). And finally, at the genus level, *Candidatus Portiera* and *Rickettsia* were the most abundant, with smaller contributions from *Candidatus Hamiltonella*, *Candidatus Cardinium*, and *Candidatus Hemipteripilus* (Figure 4D).

During October and November, bacterial communities at Bangalore Rural were dominated by *Candidatus Portiera* (94%), with *Rickettsia* contributing an additional 5% (Supplementary Figure S2A). In Chikkaballapur, *Candidatus Portiera* constituted 64% of the bacterial population, with *Rickettsia* at 28%, and smaller proportions of *Candidatus Hamiltonella* (5%), *Candidatus Hemipteripilus* (2%), and *Candidatus Cardinium* (1%) (Supplementary Figure S2B). In Kolar, *Candidatus Portiera* made up 48%, *Rickettsia* 35%, and *Candidatus Hamiltonella* 15% (Supplementary Figure S2C).

For the December–January period, *Candidatus Portiera* was dominant at Bangalore Rural (87%), followed by *Rickettsia* (11%) (Supplementary Figure S3A). At Chikkaballapur, *Candidatus Portiera* constituted 50% of the population, with *Rickettsia* at 39% and *Candidatus Hamiltonella* at 7% (Supplementary Figure S3B). In Kolar, *Candidatus Portiera* was 50%, *Rickettsia* 35%, and *Candidatus Hamiltonella* 15% (Supplementary Figure S3C). In Bangalore rural, during February through April, *Candidatus Portiera* dominated at 99% (Supplementary Figure S4A), while in Chikkaballapur, it made up 58%, with *Rickettsia* at 38% and *Candidatus Hamiltonella* at 10% (Supplementary Figure S4B). At Kolar, *Candidatus Portiera* was 48%, *Rickettsia* 35%, and *Candidatus Hamiltonella* 16% (Supplementary Figure S4C).

Seasonal fluctuations in whitefly densities were strongly influenced by weather parameters, with higher maximum temperatures correlating positively with disease severity (*r* = 0.61 to 0.84*), while minimum temperature, relative humidity, and rainfall negatively impacted both whitefly populations and ToLCuD incidence. During the Rabi season of 2020–21, whitefly populations peaked at 1.47 per 10 plants, with ToLCuD incidences reaching 13.49%, whereas the summer season of 2021 saw higher pest densities (2.08 per 10 plants) and a corresponding disease spike of 30.01%, highlighting the importance of favorable temperature regimes. Similarly, the cooler and wetter conditions in Kharif season demonstrated reduced pest and disease incidences. Higher temperatures not only supported increased whitefly populations but also favored the shifts in endosymbiont composition which affects the whitefly fitness and vectoring efficiency. The *Candidatus Portiera* dominating across all locations and seasons, accounting for 48–99% of the microbial composition; *Rickettsia* being the second-most prevalent endosymbiont and other minor players like *Candidatus Hamiltonella* (5–39%) appearing in lower proportions (5–15%), showed location-specific variations. Regression models demonstrated that weather variables accounted for 80–99% of the variability in ToLCuD prevalence, indicating that the combined effects of climatic conditions and endosymbiont-mediated vector potential drive disease outbreaks. In a nutshell, the incidence of ToLCuD across six locations in Bangalore Rural, Chikkaballapur, and Kolar districts of Karnataka was shaped as follows: the fluctuations in whitefly population dynamics, influenced by weather variables, combined with the impact of endosymbiont bacterial communities, collectively determined the patterns of disease spread.

### Metagenomics data validation using species-specific primers

**Figure 5:**
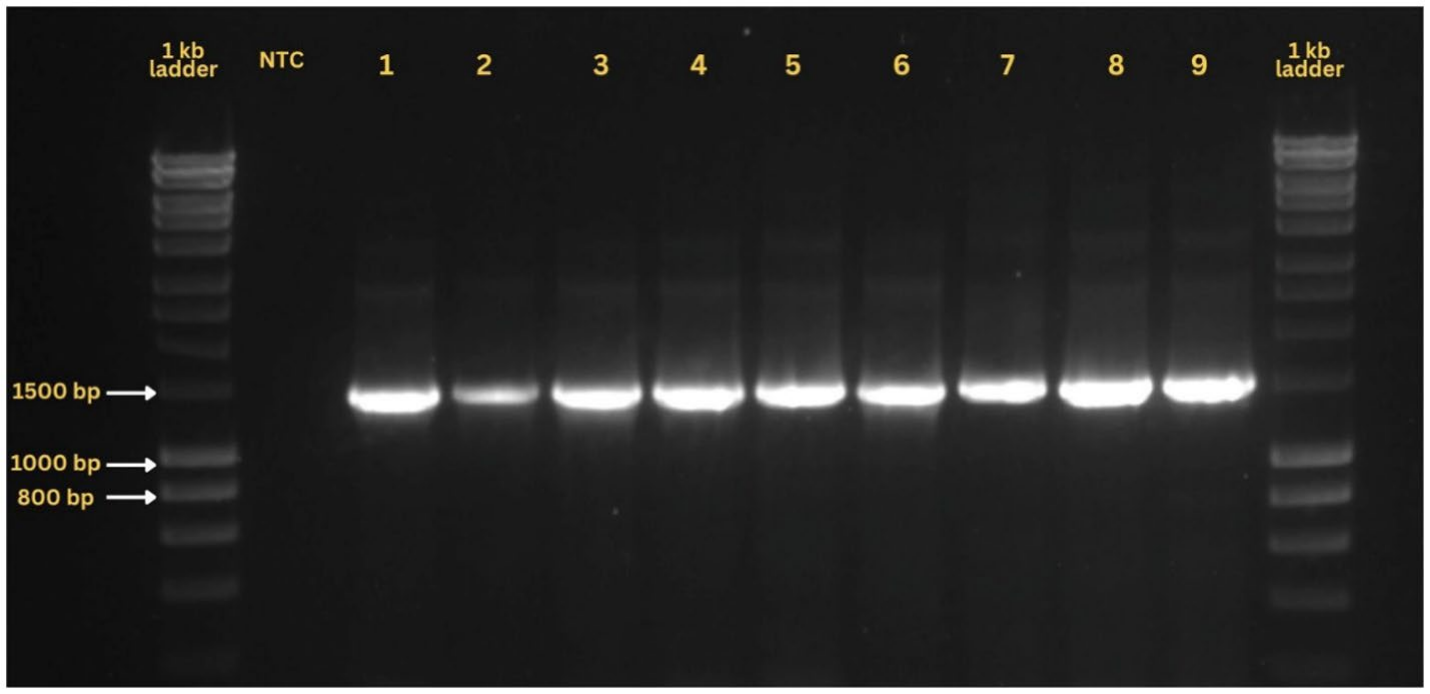
A representative ethidium bromide-stained agarose gel showing PCR amplification of the 16S rRNA gene from nine samples utilized in metagenomic analysis.

PCR validation of metagenomic samples using endosymbiont-specific primers successfully yielded expected amplicons, confirming the presence of these endosymbionts in the samples (Figure 5). No amplification was observed in negative controls across all assays, ensuring the reliability of the results. The analysis detected *Portiera*, *Cardinium*, *Hamiltonella*, *Rickettsia*, and *Wolbachia* in varying intensities across all nine samples. Details on the presence, absence, and co-occurrence of these endosymbionts are summarized in Supplementary Table S5.

## Discussion

The whitefly, *B. tabaci*, a polyphagous pest, inflicts significant damage on diverse crops due to its intraspecific complexity. Since their identification in the 1950s, *B. tabaci* biotypes, also referred to as cryptic species, genetic variants, or haplotypes have been recognized for their genetic and physiological differences, including variations in disease transmission, host range, and adaptability (Bird and Maramorosch, 1978; Ahmed et al., 2009). The biotypification of *B. tabaci* is crucial in the escalation of diseases to epidemic level (Banks et al., 2001). For example, the increasing whitefly populations on tomato crops are exacerbating the prevalence of ToLCuD, highlighting the urgent need for targeted research. While existing studies have explored the impact of cropping patterns and climate conditions on whitefly dynamics, localized insights are necessary to develop effective control strategies. This study aims to address this gap by examining the seasonal fluctuations of whitefly populations, the associated rise in ToLCuD incidence, and their relationship with endosymbionts from a metagenomic perspective.

Insects exhibit sensitivity to meteorological variations (Dostálek et al., 2018). Climatic variables such as sunshine duration, maximum and minimum temperatures, relative humidity (both maximum and minimum), and wind speed are known to influence the population dynamics of the whitefly, *B. tabaci*. In this study, all examined locations demonstrated a positive correlation between whitefly population and maximum temperature, while showing negative correlations with minimum temperature and both maximum and minimum relative humidity. This aligns with the understanding that whitefly egg and adult development are influenced cumulatively by temperature (Verma et al., 1990).

Our findings, encompassing vector population and ToLCuD incidence over the year across three districts *viz.,* Bangalore Rural, Chikkaballapur, Kolar corroborate previous research. Sharma et al. (2017) and other studies have documented that whitefly populations increase with rising temperatures, while humidity levels play a critical role in their regulation. Our study confirmed that maximum temperatures positively affect whitefly population, whereas relative humidity negatively influences them. This relationship significantly impacts the spread of ToLCuD, a devastating disease affecting tomato crops.

The conducive conditions created by continuous tomato cultivation and high temperatures facilitate the proliferation of whiteflies and the transmission of ToLCuD. Historical data shows substantial yield losses due to ToLCuD, ranging from 70% during February–May (Saikia and Muniyappa, 1989) to up to 96.90% in autumns, with severe cases reaching 100% loss (Varma and Malathi, 2003; Singh et al., 2016). Reddy et al. (2011) noted that ToLCV prevalence was widespread across Belgaum, Dharwad, and Haveri districts in Karnataka, with disease incidence varying from 4 to 100% in the Rabi season and 60 to 100% in the summer. The reliance on whitefly populations for ToLCuD transmission resulted in disease incidences ranging from 6 to 38% during winter and from 25 to 86% during summer months (Reddy, 1978).

This interplay of continuous tomato cultivation, high temperatures, suitable host plants, and viral inoculum creates an ideal environment for whitefly proliferation and ToLCuD spread (Banks et al., 2001; Singh et al., 2016). The presence of diverse cryptic species of *B. tabaci*, which efficiently transmit the virus, further complicates the situation. These findings underscore the need for comprehensive management strategies to control both whitefly population and ToLCuD, thereby safeguarding tomato crops and supporting farmer livelihoods.

When exploring the drivers behind the vector efficiency of whiteflies, it becomes evident that temperature alone does not encapsulate the complexity of their biology. The discovery of endosymbionts reveals an additional dimension of this intricate relationship. This study investigates the bacterial communities associated with two cryptic species of whitefly, Asia II-5 and Asia II-7, infesting tomato plants in the Eastern dry zones of Karnataka, India. By examining how these endosymbiont communities vary across different months and geographical locations, we aim to uncover their role in the vector dynamics of whiteflies.

*Bemisia tabaci* harbors a diverse array of endosymbiotic bacteria within its gut, which contribute to various functions ranging from polyphagy to overall fitness, including survival, adaptation, and evolution (Su et al., 2015; Upadhyay et al., 2015; Chen et al., 2016; Liu et al., 2016; Zhang et al., 2016). Historically, studies on insect symbiotic microbes were constrained by traditional isolation and culture techniques, limiting functional analysis of unculturable microbes. However, advancements in high-throughput metagenomic sequencing have enabled efficient functional characterization of these symbiotic bacteria. This next-generation sequencing approach surpasses traditional primer-based amplification methods, providing a comprehensive view of microbial diversity and facilitating the exploration of insect microbiomes (Lv et al., 2018; Su et al., 2016).

In this study, metagenomic DNA from *B. tabaci* cryptic species was extracted and sequenced using the Illumina Seq NGS Platform, focusing on the amplification of the 16SrRNA gene (Chiel et al., 2007; Gueguen et al., 2010). The analysis revealed a rich diversity of bacterial communities, with 1,219 Amplicon Sequence Variants (ASVs), of which 92% were assigned to specific genera and 8% remained unclassified. *Proteobacteria* emerged prominently at the phylum level, encompassing microbes ranging from parasitic and pathogenic to mutualistic forms. Within *Proteobacteria*, the samples were dominated by *Alphaproteobacteria*, known for their role as facultative symbionts, and *Gammaproteobacteria*, which provide essential nutrients to arthropods (Matsuura et al., 2012; Serrato-Salas and Gendrin, 2023).

The primary endosymbiont identified was *Candidatus Portiera*, while secondary endosymbionts included *Wolbachia* (Rickettsiales), *Rickettsia* (Rickettsiales), *Cardinium* (Bacteroidetes), and *Arsenophonus* (Enterobacteriales). These findings align with previous research (Bing et al., 2013; Gottlieb et al., 2006; Hashmi et al., 2018; Jahan et al., 2015), which documented a similar suite of endosymbionts. Notably, *Rickettsia*, along with *Candidatus Cardinium* and *Wolbachia*, was found in higher abundance compared to other secondary endosymbionts.

At the genus level, *Candidatus Portiera* and *Rickettsia* were consistently predominant across all samples, with traces of *Candidatus Hamiltonella*, *Candidatus Cardinium*, and *Candidatus Hemipteriphilus*. This diversity in endosymbiotic bacteria is likely influenced by seasonal variations, weather conditions (temperature, precipitation, and relative humidity), and agricultural practices, which collectively affect whitefly populations within their ecosystem.

The primary endosymbiont *Portiera* is crucial for the host’s survival, while secondary endosymbionts play roles in enhancing population resilience under varying conditions. They contribute to increased egg and nymph survival, accelerated reproduction, larger body size, heat tolerance, and resistance to insecticides and parasitoids, thereby supporting the co-evolution of whiteflies with their host plants (Hedges et al., 2008; Oliver et al., 2005; Teixeira et al., 2008; Tsuchida et al., 2004; Su et al., 2013).

The Principal Coordinates Analysis (PCoA) revealed that microbial communities from locations of Kolar and Chikkaballapur districts exhibit greater similarity to each other compared to those from Bangalore Rural. This observation suggests that the microbial assemblages in Kolar and Chikkaballapur may be influenced by distinct selection pressures, including different management practices, natural predators, and environmental conditions. Furthermore, the Shannon and Simpson diversity indices were notably higher in samples from Chikkaballapur and Kolar districts than those from Bangalore Rural. This disparity might be attributed to the more intensive vegetable cultivation in these areas, which promotes whitefly movement between crops, coupled with variations in local weather conditions.

Previous research has demonstrated that host plants and geographic factors influence symbiont frequencies in *B. tabaci* (Pan et al., 2012), the pea aphid *Acrythosiphon pisum* (Harris) (Tsuchida et al., 2002), and the psyllid *Glycaspis brimblecombei* (Moore), with prevalence often correlating with climatic variables. These findings suggest that environmental factors, including geographic location, agricultural practices, and natural enemies, collectively exert significant selection pressures that drive the dynamics of natural symbiont populations (Cheng et al., 2017; Gressel, 2018; Vijayakumar et al., 2018; Xie et al., 2018).

In this study of whiteflies, *Proteobacteria* emerged as the predominant bacterial taxon, followed by *Firmicutes* and *Actinobacteria*. This finding aligns with previous research by (Jones et al., 2013), which identified *Proteobacteria* as the dominant bacterial group in mosquito guts. This phylum is also prevalent in various insects, including whiteflies (Jones et al., 2013; Osei-Poku et al., 2012; Wang et al., 2011; Su et al., 2016). Conversely, *Firmicutes* and *Actinobacteria* are the major bacterial phyla in the midgut of *Helicoverpa armigera* (Hubner) larvae (Gayatri Priya et al., 2012) and the reproductive tissues of *Bactrocera minax* (Dacuct) (Lundgren et al., 2007).

In whiteflies, endosymbionts, particularly *Portiera aleyrodidarum*, contribute essential nutrients such as B-complex vitamins and amino acids, which are deficient in their diet, and aid in carbohydrate degradation and pesticide detoxification (Delalibera et al., 2005; Werren, 2012; Xie et al., 2018). Additionally, endosymbionts play a role in virus transmission by protecting the virus during its passage through the haemolymph, with a GroEL homolog facilitating circulative transmission (Morin et al., 1999; Rana et al., 2012). Unlike primary endosymbionts (PEs), secondary endosymbionts (SEs) are not restricted to bacteriocytes and can inhabit various tissues, including salivary glands, malphigian tubules, and reproductive organs, without significantly impacting host survival upon removal (Xue et al., 2012; Su et al., 2014). However, in some hemipterans, SEs complement PE functions and establish a closer, co-obligate association with the host (Manzano-Marín and Latorre, 2014).

Secondary endosymbionts, such as *Rickettsia*, have been identified as significant factors influencing the susceptibility of whiteflies to commonly used insecticides like pyriproxyfen, acetamiprid, spiromesifen, and thiamethoxam (Kontsedalov et al., 2008). *Rickettsia* in the MED biotype of *B. tabaci* in Israel enhances the transmission of ToLCuD by inducing stress and immune responses that confer heat tolerance and viral resistance (Gottlieb et al., 2006). Similarly, *Hamiltonella* found in the B-biotype (currently referred as MEAM cryptic species) whiteflies facilitates successful TYLCV transmission (Gottlieb et al., 2010), while *Arsenophonus* supports the Asia II cryptic species in transmitting cotton leaf curl virus (Rana et al., 2012). The efficiency of TYLCV transmission varies among biotypes harboring *Hamiltonella*, highlighting its potential as a novel pest control target and indicator of pest status. Notably, biotypes like JPL, Asia II-6, and MED Q2, which lack *Hamiltonella*, exhibit reduced TYLCV transmission efficiency and increased sensitivity to pesticides (Kijima et al., 2012; Ueda et al., 2009).

In contrast, *Hamiltonella* has been reported to confer virus resistance in pea aphids (Oliver et al., 2003). *Arsenophonus* and *Cardinium* are more prevalent in cryptic species from Asia compared to MED and MEAM cryptic species. Additionally, *Wolbachia* is implicated in cytoplasmic incompatibility phenomena in *B. tabaci*, as evidenced by numerous reports of mating incompatibilities among different populations and biotypes (Bedford et al., 1994; Brown et al., 1995; Byrne et al., 1995; De Barro and Hart, 2000). Despite the lack of controlled crossing tests between strains with identical nuclear backgrounds and infections, *Wolbachia* is thought to contribute to the evolution of MED cryptic species, aiding in parasitoid resistance and supporting the role of endosymbionts in speciation (Bordenstein et al., 2001; De Barro et al., 2000; Frohlich et al., 1999; Shoemaker et al., 1999; Xue et al., 2012). The current study underscores that these antagonistic and synergistic interactions among endosymbionts in whiteflies reflect a complex tripartite relationship, where endosymbionts vie for host resources and molecular machinery.

Endosymbionts significantly influence host fitness and reproduction, paralleling the effects of environmental conditions such as weather. The interaction between these effects-whether they act independently or complementarity remains an area of active investigation. One intriguing hypothesis is that endosymbionts might modulate host reproduction through mechanisms like cytoplasmic incompatibility or male feminization, similar to phenomena observed in parasitoid wasps of the genus, *Encarsia* (Engelstädter and Hurst, 2009). This is particularly plausible given the close phylogenetic relationship between *Cardinium* strains in *B. tabaci* and those found in *Encarsia*, reflecting a parasitoid-host dynamic that facilitates the exchange of bacterial symbionts (Santos-Garcia et al., 2014).

Currently, the best-documented mechanism involves endosymbiont proteins such as GroEL proteins, which protect plant viruses by binding to their coat proteins, thereby shielding the virus from the host’s immune defenses. The present study leveraged metagenomic approaches to investigate endosymbiont diversity in *B. tabaci* population across Karnataka, examining the interplay between meteorological factors, whitefly population dynamics, and TYLCV incidence. High-throughput sequencing technologies have minimized false negatives associated with PCR-based detection, providing a more comprehensive view of the microbial communities associated with whiteflies.

As we delve into these findings, it becomes evident that further research is essential. A deeper exploration of how endosymbionts impact host fitness and reproduction could reveal novel insights into ecological interactions and evolutionary processes. Understanding these dynamics may uncover new facets of how endosymbionts and environmental factors shape the biology and adaptability of whiteflies.

## Conclusion

This study thoroughly examined the population dynamics of *B. tabaci* and the incidence of ToLCuD correlating with weather factors and associated endosymbionts across principal tomato-growing parts of Karnataka. Multiple Regression analysis depicted a positive correlation between whitefly population and the virus incidence. Seasonal patterns were evident, with peak populations and disease incidence recorded during the summer, driven by higher temperatures, reduced humidity, and lower rainfall. Compared to Chikkaballapur and Bangalore Rural, Kolar district recorded the highest whitefly population, demonstrating significant spatial and temporal variation. Molecular characterization revealed the presence of Asia II-5 and Asia II-7 cryptic species, with Asia II-5 predominating in most regions, highlighting its potential role in vectoring ToLCuD. Metagenomic analyses using the 16S rRNA gene identified diverse bacterial communities dominated by *Candidatus Portiera*, *Rickettsia*, and *Candidatus Hamiltonella*. These symbionts exhibited notable seasonal and location-specific shifts, suggesting their influence on whitefly biology and disease transmission. These findings were validated through PCR using endosymbiont-specific primers on samples collected from different regions of Karnataka. Overall, our findings highlight the complex interactions between whitefly populations, their microbiome, and environmental factors, stressing the need for targeted, season-specific pest and disease management strategies in high-risk areas. To fully grasp the dynamics of endosymbiont variation within these cryptic species across three distinct districts in Karnataka, further regional studies and detailed analyses are imperative. Future research should focus on the functional roles of these endosymbionts in enhancing the vector’s efficiency and invasiveness. A comprehensive understanding of these endosymbionts could pave the way for innovative pest management strategies and improved utilization of beneficial insects through collaborative research.

## Data Availability

The data generated from this Metagenome Sequencing project was deposited in the NCBI Sequence Read Archive (SRA) under the BioProject accession ID: PRJNA1187347. The corresponding SRA accession numbers for the nine samples are SRR31381468, SRR31381469, SRR31381470, SRR31381471, SRR31381472, SRR31381473, SRR31381474, SRR31381475, and SRR31381476.
