# Supplementary material for: Influence of weather and seasonal factors on whitefly dynamics, associated endosymbiotic microbiomes, and Begomovirus transmission causing tomato leaf curl disease: insights from a metagenomic perspective
Source: Front Microbiol. 2025 Mar 12;16:1555058. doi: 10.3389/fmicb.2025.1555058 (PMC11936956; doi:10.3389/fmicb.2025.1555058)
Supplement: Supplementary file 1 [file Data_Sheet_1.pdf]

Supplementary Tables:

Table S1: Pooling of DNA samples from three districts

Sample name	Month	District	Location
**ONCHI**	October-November	Chikkaballapur	Doddaganjur + Kencharlahalli
**ONKOL**	October- November	Kolar	Kalluru + Srinivaspur
**ONBRU**	October- November	Bangalore Rural	Tippuru+ Kundana
**DJCHI**	December-January	Chikkaballapur	Doddaganjur + Kencharlahalli
**DJKOL**	December-January	Kolar	Kalluru + Srinivaspur
**DJBRU**	December-January	Bangalore Rural	Tippuru + Kundana
**FMACHI**	February-April	Chikkaballapur	Doddaganjur + Kencharlahalli
**FMAKOL**	February-April	Kolar	Kalluru + Srinivaspur
**FMABRU**	February-April	Bangalore Rural	Tippuru + Kundana

Table S2: Population dynamics of whiteflies and percentage incidence of tomato leaf curl disease in major tomato-growing areas

Season	Month	SMW	Population of whitefly/10 plants							Incidence of tomato leaf curl disease (%)						
			D. Ganjur	K. halli	Kalluru	S. Pura	Tippuru	Kundana	Mean	D. Ganjur	K. halli	Kalluru	S. Pura	Tippuru	Kundana	Mean
Rabi 2020-2021	Oct.	44	0.00	0.29	0.60	0.99	0.13	0.00	**0.33**	0.00	3.00	4.20	5.00	0.00	0.00	**2.03**
	Nov.	47	0.70	0.90	1.61	1.90	0.65	0.49	**1.04**	5.80	6.90	8.30	9.98	5.70	5.40	**7.01**
	Dec.	52	1.47	1.90	3.07	2.80	1.40	1.25	**1.98**	13.49	15.34	17.89	18.02	13.02	12.24	**15.00**
	Jan.	4	1.34	1.50	2.53	3.20	1.30	1.32	**1.86**	11.20	11.60	17.30	17.99	10.92	10.70	**13.28**
Summer 2021	Feb.	8	1.20	1.00	2.34	2.45	0.89	1.02	**1.48**	10.50	11.00	15.00	17.00	10.50	10.56	**12.42**
	Mar.	12	2.08	3.40	5.20	6.02	1.95	1.89	**3.42**	30.01	34.20	36.08	38.90	27.15	25.12	**31.91**
	Apr.	17	2.00	3.10	5.14	5.80	1.93	1.87	**3.30**	29.92	33.00	36.10	37.70	26.99	25.02	**31.45**
Kharif 2021	Jul.	30	0.50	0.76	1.25	2.10	0.53	0.32	**0.91**	0.00	4.25	3.00	4.80	0.00	3.30	**2.55**
	Aug.	35	1.02	1.32	1.50	2.35	1.20	1.00	**1.39**	8.60	10.54	11.51	12.81	8.40	8.18	**10.00**
	Sep.	39	1.30	1.88	2.39	2.90	1.29	1.18	**1.82**	10.60	12.30	12.80	13.04	10.20	10.00	**11.49**
Mean			1.16	1.60	2.56	3.05	1.12	1.03	**1.75**	12.01	14.21	16.21	17.52	11.28	11.05	**13.71**

SMW-Meteorological Standard Week

Table S3: Correlation and regression analysis using multiple linear regression between meteorological parameters, ToLCuD incidence, and *B. tabaci* population across three districts

District	Locations (Regions)	Temperature (°C)		Relative humidity (%)		Rainfall (mm) (X5)	Whitefly population	R^2	Regression equation
		T max. (X1)	T min. (X2)	Max. (X3)	Min. (X4)		(X6)		
Chikkaballapur	Doddaganjur	0.63	-0.32	-0.02	-0.84*	-0.39	-0.34	0.82	Y = 20.77 +1.90X1-0.85X2 0.96X3+0.33X4+0.01 X5-0.13X6
	Kencharlahalli	0.84*	-0.11	-0.83*	-0.78*	-0.58	0.97*	0.98	Y = -69.94 +3.24X1-3.37X2 0.14X3+0.55X4+0.06X5+9.11X6
Kolar	Kalluru	0.61	-0.29	-0.06	-0.81*	-0.40	-0.28	0.80	Y = 126.35 -2.18X1+5.23X2-1.24 X3-0.47 X4-0.10X5-1.01 X6
	Srinivaspura	0.81*	-0.24	-0.83*	-0.75*	-0.66*	0.97*	0.99	Y = 33.31 +0.57X1-3.45X2- 0.75X3+0.81X4+0.06X5+8.62X6
Bangalore Rural	Tippuru	0.62	-0.25	-0.29	-0.76*	-0.37	-0.35	0.96	Y = -757.07 +16.79X1-5.38X2 +2.64X3+2.57X4+0.03 X5+2.70X6
	Kundana	0.83*	-0.01	-0.64*	-0.87*	-0.57	0.95*	0.98	Y= -89.73 +2.17X1-0.13X2+ 0.30X3+0.12X4-0.02X5+7.99X6

Table S4: Total number of paired end reads obtained for *B. tabaci* samples before and after applying standard filtration parameters using the *filterandTrim* function in both forward and reverse orientations

Sample Name	Before filtration			After filtration			Read loss in percent (%)
	Forward	Reverse	combined	Forward	Reverse	Combined	
**DJBRU**	97691	97691	**195382**	87201	87201	**174402**	**10.73**
**DJCHI**	112199	112199	**224398**	99773	99773	**199546**	**11.07**
**DJKOL**	111629	111629	**223258**	100877	100877	**201754**	**9.63**
**FMABRU**	100063	100063	**200126**	86653	86653	**173306**	**13.40**
**FMACHI**	72589	72589	**145178**	64333	64333	**128666**	**11.37**
**FMAKOL**	103897	103897	**207794**	92524	92524	**185048**	**10.94**
**ONBRU**	93611	93611	**187222**	83575	83575	**167150**	**10.72**
**ONCHI**	95969	95969	**191938**	87249	87249	**174498**	**9.08**
**ONKOL**	100766	100766	**201532**	90999	90999	**181998**	**9.69**

Table S5: Primary and secondary endosymbionts identified from *B. tabaci* using species specific primers

Sample name	Month	Location	*Portiera*	*Arsenophonus*	*Cardinium*	*Hamiltonella*	*Rickettsia*	*Wolbachia*
**ONCHI**	October -November	Chikkaballapur	√	×	√	√	√	√
**ONKOL**	October- November	Kolar	√	×	√	√	√	√
**ONBRU**	October- November	Bangalore Rural	√	√	√	×	√	×
**DJCHI**	December- January	Chikkaballapur	√	√	√	√	√	√
**DJKOL**	December- January	Kolar	√	√	√	√	√	×
**DJBRU**	December- January	Bangalore Rural	√	×	√	×	√	×
**FMACHI**	February-April	Chikkaballapur	√	√	√	√	√	√
**FMAKOL**	February- April	Kolar	√	√	√	√	√	√
**FMABRU**	February- April	Bangalore Rural	√	√	√	×	√	×

√= Presence of Endosymbiont, ×= Absence of Endosymbiont

Supplementary Figures:

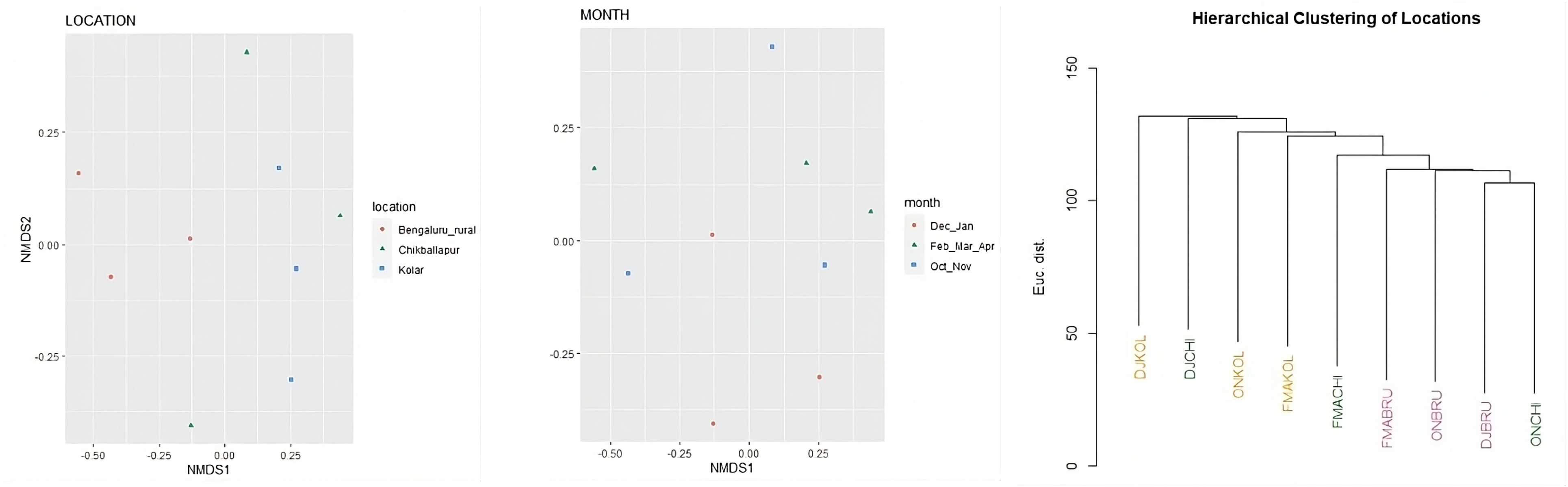

(A) **(B)** **(C)**

Figure S1: Beta Diversity Analysis Plots: (A) Principal Coordinate Analysis (PCoA) based on location; (B) Principal Coordinate Analysis (PCoA) based on month; (C) Hierarchical clustering of samples using Euclidean distance metric

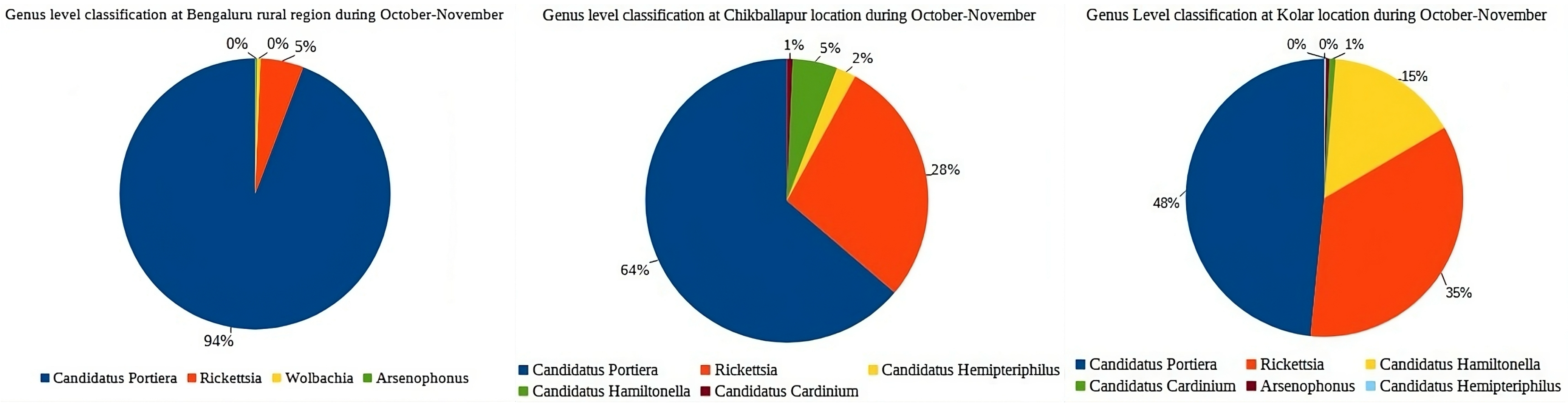

(A) (B) (C)

Figure S2: Genus-level classification of samples collected during October-November: (A) Sampling at the Bangalore rural location; (B) Sampling at the Chikkaballapur location; (C) Sampling at the Kolar location

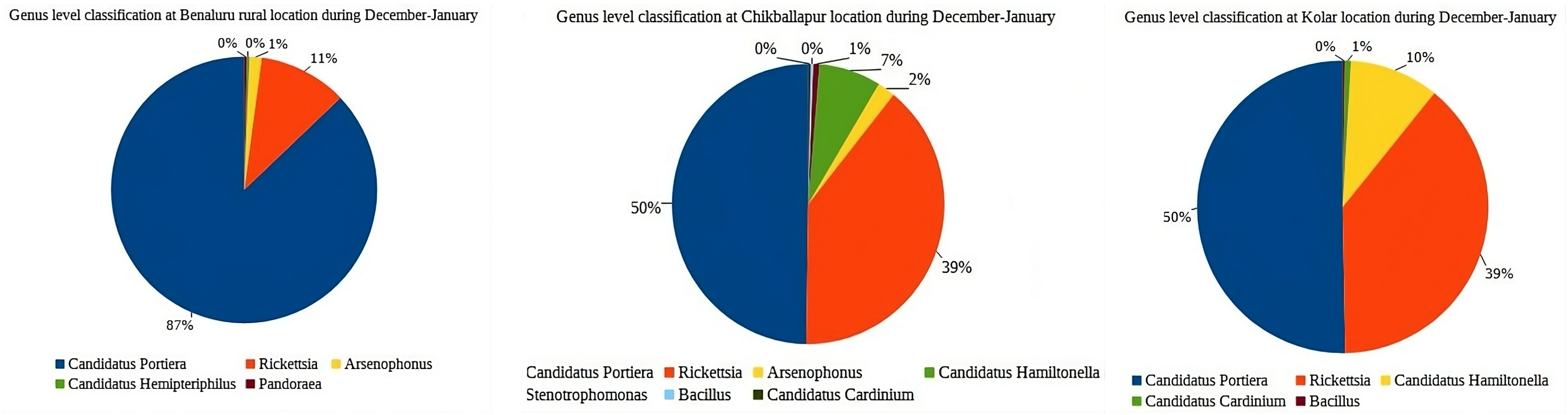

(A) **(B)** **(C)**

Figure S3: Genus-level classification of samples collected during December-January: (A) Sampling at the Bangalore rural location; (B) Sampling at the Chikkaballapur location; (C) Sampling at the Kolar location

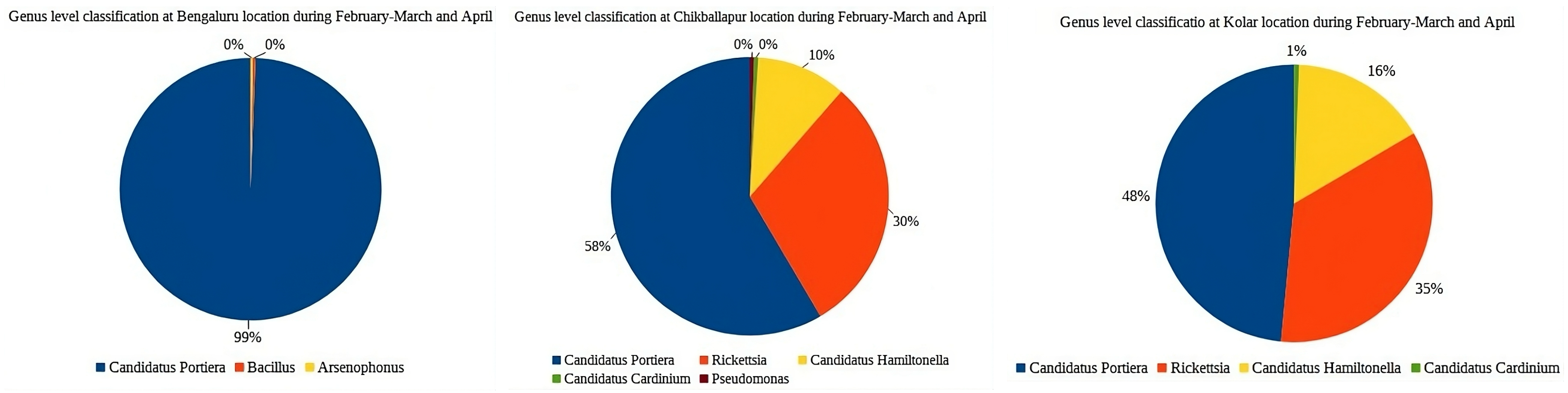

(A) **(B)** **(C)**

Figure S4: Genus-level classification of samples collected during February-March and April: (A) Sampling at the Bangalore rural location; (B) Sampling at the Chikkaballapur location; (C) Sampling at the Kolar location
