# Supplementary figures and images for: Influence of weather and seasonal factors on whitefly dynamics, associated endosymbiotic microbiomes, and Begomovirus transmission causing tomato leaf curl disease: insights from a metagenomic perspective
Source: Front Microbiol. 2025 Mar 12;16:1555058. doi: 10.3389/fmicb.2025.1555058 (PMC11936956; doi:10.3389/fmicb.2025.1555058)

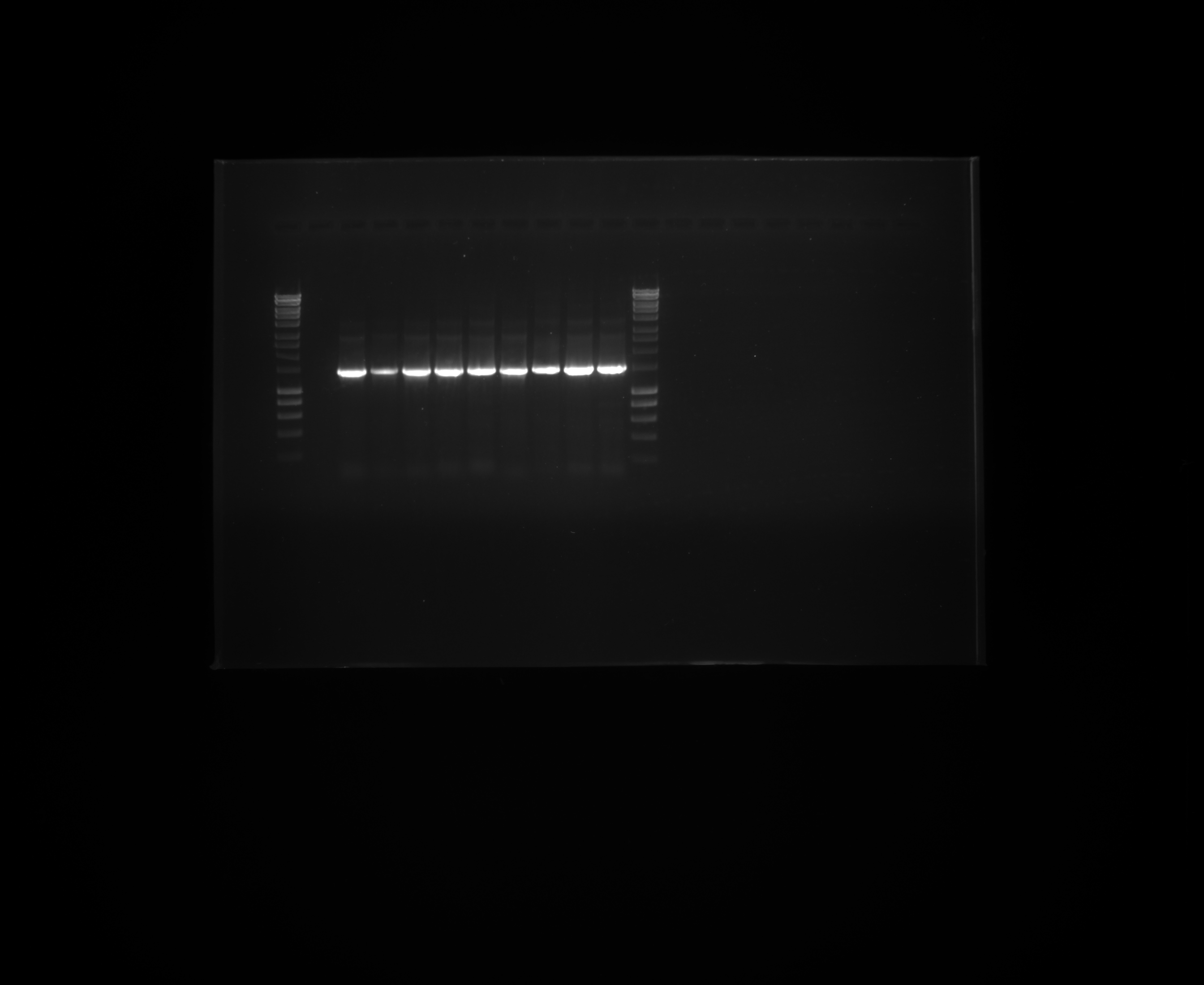

Supplement: Supplementary file 2 [file Image_1.jpeg]
